# Discovery of GABA Aminotransferase Inhibitors via Molecular Docking, Molecular Dynamic Simulation, and Biological Evaluation

**DOI:** 10.3390/ijms242316990

**Published:** 2023-11-30

**Authors:** Muhammad Yasir, Jinyoung Park, Yuno Lee, Eun-Taek Han, Won Sun Park, Jin-Hee Han, Yong-Soo Kwon, Hee-Jae Lee, Wanjoo Chun

**Affiliations:** 1 Department of Pharmacology, Kangwon National University School of Medicine, Chuncheon 24341, Republic of Korea; yasir.khokhar1999@gmail.com (M.Y.); jinyoung0326@kangwon.ac.kr (J.P.); heejaelee@kangwon.ac.kr (H.-J.L.); 2 Drug Information Platform Center, Korea Research Institute of Chemical Technology, Daejeon 34114, Republic of Korea; yunolee1@krict.re.kr; 3 Department of Medical Environmental Biology and Tropical Medicine, Kangwon National University School of Medicine, Chuncheon 24341, Republic of Korea; ethan@kangwon.ac.kr (E.-T.H.); han.han@kangwon.ac.kr (J.-H.H.); 4 Department of Physiology, Kangwon National University School of Medicine, Chuncheon 24341, Republic of Korea; parkws@kangwon.ac.kr; 5 College of Pharmacy, Kangwon National University School of Medicine, Chuncheon 24341, Republic of Korea

**Keywords:** GABA-AT, pharmacophore, molecular docking, molecular dynamic simulation, gmx_MMPBSA

## Abstract

γ-Aminobutyric acid aminotransferase (GABA-AT) is a pyridoxal 5′-phosphate (PLP)-dependent enzyme that degrades γ-aminobutyric (GABA) in the brain. GABA is an important inhibitory neurotransmitter that plays important neurological roles in the brain. Therefore, GABA-AT is an important drug target that regulates GABA levels. Novel and potent drug development to inhibit GABA-AT is still a very challenging task. In this study, we aimed to devise novel and potent inhibitors against GABA-AT using computer-aided drug design (CADD) tools. Since the crystal structure of human GABA-AT was not yet available, we utilized a homologous structure derived from our previously published paper. To identify highly potent compounds relative to vigabatrin, an FDA-approved drug against human GABA-AT, we developed a pharmacophore analysis protocol for 530,000 Korea Chemical Bank (KCB) compounds and selected the top 50 compounds for further screening. Preliminary biological analysis was carried out for these 50 compounds and 16 compounds were further assessed. Subsequently, molecular docking, molecular dynamics (MD) simulations, and binding free energy calculations were carried out. In the results, four predicted compounds, A07, B07, D08, and H08, were found to be highly potent and were further evaluated by a biological activity assay to confirm the results of the GABA-AT activity inhibition assay.

## 1. Introduction

Epilepsy is a neurological disorder characterized by recurrent unprovoked seizures, which are abnormal electrical discharges in the brain [1,2]. GABA (gamma-aminobutyric acid), an important neurotransmitter in the brain that plays a significant role in regulating the balance of excitation and inhibition [3,4,5], is synthesized from glutamate within presynaptic GABAergic neurons. The release of GABA for neurotransmission is triggered by the depolarization of these presynaptic neurons [6,7]. Upon its release into the synaptic cleft, GABA has the capacity to attach to one of the two primary GABA receptors found on postsynaptic neurons, namely GABA_A_ and GABA_B_ receptors. When GABA binds allosterically to GABA_A_ receptors, it triggers the opening of the central chloride ion channel within the receptor [8]. This, in turn, leads to hyperpolarization of the neuronal membrane, reducing cell excitability and, as a result, promoting neuronal inhibition [9,10]. GABA_B_ receptors function as metabotropic receptors, and their activation by GABA leads to G-protein coupled receptor-mediated opening of associated potassium channels. This process, in turn, leads to hyperpolarization and neuronal inhibition, similar to the effects observed with GABA_A_ receptors [11]. Following synaptic neurotransmission, GABA’s activity is concluded through its reuptake, which can occur either by being taken back up into the presynaptic neurons or into glial cells, facilitated by GABA transporters [12,13,14]. Within glial cells, GABA undergoes catabolism through the action of an enzyme known as GABA-AT, which is also referred to as GABA-transaminase (GABA-T), 4-aminobutyrate transaminase, or 4-aminobutyrate aminotransferase [15,16].

GABA-AT belongs to an extensive family of pyridoxal 5′-phosphate (PLP)-dependent aminotransferases and is responsible for facilitating the breakdown of GABA into succinic semialdehyde [17,18,19]. In the course of this enzymatic process, the coenzyme PLP, which is bound to Lys329 (or Lys357 in humans), undergoes conversion to pyridoxamine 5′-phosphate (PMP). As a result, when PLP is transformed into PMP, the enzyme temporarily loses its catalytic activity since it has been altered. To regain its catalytic functionality, a subsequent catalytic step is necessary in which the enzyme utilizes a second substrate, α-ketoglutarate, to convert PMP back to PLP, enabling catalysis to recommence [20]. Consequently, as a result of this process, α-ketoglutarate undergoes conversion into the excitatory neurotransmitter glutamate [21]. Hence, in the overall process, one molecule of GABA is transformed into one molecule of glutamate. The mechanism responsible for converting PMP back to PLP is the reverse of the mechanism that transforms PLP into PMP. The PLP-dependent enzyme known as glutamic acid decarboxylase (GAD) plays a pivotal role in catalyzing the conversion of glutamate into GABA. Therefore, the interplay between the two PLP-dependent enzymes, GABA-AT and GAD, is crucial for regulating the levels of these two neurotransmitters in the brain [22]. In typical circumstances, convulsions may be induced when the concentration of GABA falls below a certain threshold in the brain, while elevating GABA levels can halt seizures. As a result, the inhibition of GABA-AT using substances like vigabatrin has demonstrated effectiveness in reducing excessive neural activity in individuals with epilepsy [23,24]. Nonetheless, in disorders related to GABA aminotransferase deficiency, where there is an abnormal increase in endogenous GABA levels due to mutations in GABA-AT, the frequency of seizures is notably heightened, leading to a condition referred to as epileptic encephalopathy [25]. The reasons behind the seizure-inducing effects of consistently elevated GABA levels in individuals with GABA-AT deficiency remain unclear. Several potential hypotheses have been suggested, such as the excessive inhibition of inhibitory interneurons, paradoxical depolarizing effects, and downregulation of GABA receptors. Similar mechanisms have been observed in another disorder related to GABA metabolism, known as succinic semialdehyde dehydrogenase deficiency [26].

This study aims to discover potent GABA-AT inhibitors using a combination of in silico and biological evaluation approaches. Through pharmacophore modeling, molecular docking, MD simulations, binding free energy calculations, and biological validation, four compounds, A07, B07, D08, and H08, were identified as being highly potent against GABA-AT.

## 2. Results and Discussion

### 2.1. Structural Analysis of GABA-AT Protein

**Figure 1:**
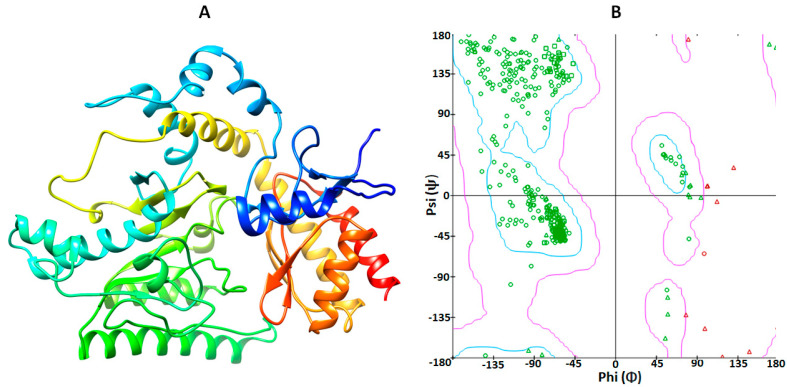
(**A**) The 3D structure of GABA-AT protein was visualized using UCSF Chimera, and (**B**) the Ramachandran plot was generated through calculations in Discovery Studio.

Human GABA-AT is a homodimeric protein consisting of 461 amino acid residues. The three-dimensional structure of human GABA protein was obtained from our previously published data on the mutational analysis of GABA-AT, in which homology modeling was carried out against the reference model of *Sus scrofa* with 1.63 Å resolution (PDBID 4Y0D) and the sequence similarity of *Sus scrofa* was depicted to be 95.67% against human GABA-AT [27]. SWISS-MODEL was employed to generate the 3D model of human GABA-AT. The overall structure of GABA-AT included loops, α-helices, and β-sheets. Furthermore, according to the VADAR 1.8 structural values, GABA-AT was composed of approximately 39% α-helices, 19% β-sheets, 40% coils, and 26% turns. Additionally, the Ramachandran plot indicated that a substantial 95.6% of amino acids were situated within the favored region, with 99.1% of residues falling into the allowed zone in terms of dihedral angles phi (φ) and psi (ψ) (Figure 1A,B).

### 2.2. Binding Pocket Analysis

**Figure 2:**
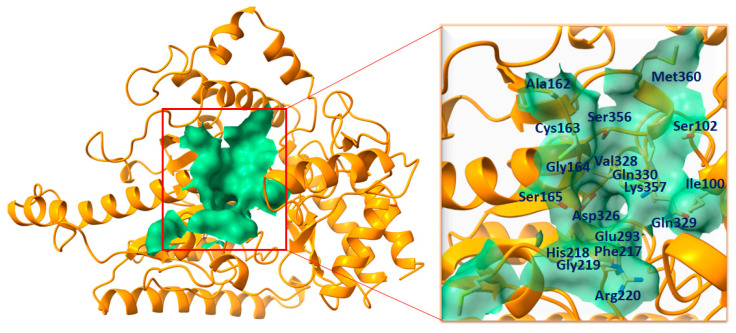
The complete structural depiction of GABA-AT is presented, featuring the binding pocket is presented on left. The entire protein is represented in dark orange, while the binding surface area is highlighted in medium sea green. While, The active site residues are indicated by their positions within the active region of the target protein and are denoted in dark blue on the right side.

The function of a binding pocket within a protein is not only determined by its structure and location but also influenced by the specific group of amino acid residues that surround it [28]. The binding pocket residues of GABA-AT were chosen from already published data [27], in which we calculated the binding pocket residues of human GABA-AT using an online webserver, PrankWeb (https://prankweb.cz/) (accessed on 25 March 2023), and selected as Ile100, Ser102, Ala162, Cys163, Gly164, Ser165, Phe217, His218, Gly219, Arg220, Glu293, Asp326, Val328, Gln_329, Gln330, Ser356, Lys357, and Met360. Moreover, the binding pocket of GABA-AT was visualized using Discovery Studio and UCSF Chimera to confirm the residual binding position (Figure 2).

### 2.3. Pharmacophore Analysis

**Figure 3:**
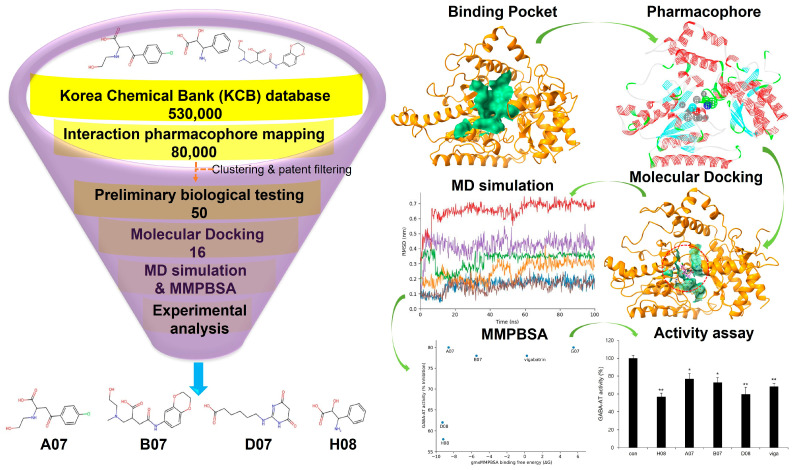
Pharmacophore analysis and overall workflow. The KCB database was selected for the screening of potent compounds against GABA-AT. After rigorous clustering and patent filtering 50 compounds were subjected to preliminary biological testing. Moreover, the selected 16 compounds were indulged to further screening by molecular docking, MD simulation, gmx_MMPBSA and biological activity. Therefore, 4 compounds A07, B07, D07, and H08 were found to be promising against GABA-AT (single (*) and double (**) marks represent statistical significance at *p* < 0.05 and *p* < 0.01, respectively).

For optimal structure-based virtual screening, it was important to create a reasonable protein–ligand pharmacophore model that could be used as a filtering query. To build this model, the adjusted structure of the binding site to the highly active compound was required. Therefore, vigabatrin was docked at the active binding site of GABA-AT protein in order to find out relatively novel scaffolds. From the given structure, a 3D protein–ligand pharmacophore model was constructed. This model consisted of one hydrophobic feature, two hydrogen bond acceptor features, one negative ionizable feature, and one positive ionizable feature. Subsequently, this pharmacophore model was employed as a 3D query to search the Korea Chemical Bank (KCB) database, which contains 530,000 compounds. Approximately 80,000 compounds were filtered out based on a fit value greater than 2.5. From this refined set, around 50 molecules were selected, taking into account their chemical diversity and patent filtering criteria (Figure 3). These chosen molecules were then subjected to preliminary biological testing to evaluate their potential biological activity (Supplementary Data Figure S1). Therefore, 16 compounds were selected for further analysis.

### 2.4. Molecular Docking Analysis

A set of 16 compounds was subjected to docking simulations against GABA-AT. The resulting docked complexes were individually assessed and scored by considering factors such as minimal docking energy and interaction energy values (Table 1). The Discovery Studio CDocker module provides two types of energy values, namely CDocker energy and CDocker interaction energy. CDocker energy represents the overall docking energy, taking into account the 3D structural and physiochemical characteristics of both the ligand and protein. On the other hand, CDocker interaction energy delves into the specifics of each interaction between the ligand and receptor. It assesses the impact of intermolecular forces, such as van der Waals forces, electrostatic interactions, and hydrogen bonds, on the overall binding strength [29,30]. D07 demonstrated the lowest CDocker energy value. Moreover, F07, B08, A07, and H08 were among the top five compounds and manifested CDocker energies of −72.1, −60.5, −59.4, and −58.5, respectively. Overall, nine screened compounds exhibited lower docking energies than the reference compound vigabatrin (−53.7).

### 2.5. Binding Interaction Analysis against GABA-AT

The interactions between the top 16 docked compounds and the GABA-AT receptor were investigated using Discovery Studio and UCSF Chimera. This analysis aimed to confirm the binding interactions of the ligands with the amino acid residues at the active site of GABA-AT. Based on the docking energy values, preliminary biological results, and good binding interactions, four compounds, A07, B07, D08, and H08, and the reference compound vigabatrin are presented in the following while the 2D depictions of all docked compounds are illustrated in Figure S2 in the Supplementary Data.

**Figure 4:**
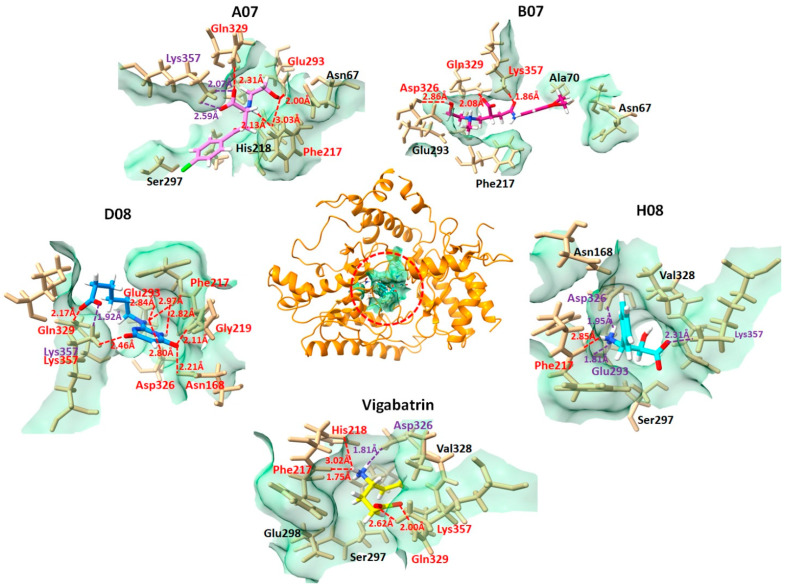
Graphical representation comparing the combined compounds A07, B08, D08, and H08 with vigabatrin in their interactions with the amino acid residues in the active region of GABA-AT. This visualization allows for the examination of how these compounds interact with the active site of GABA-AT, providing insights into their binding behavior and potential therapeutic relevance. GABA-AT protein is depicted at the center in dark orange, while the ligand interactions are illustrated in distinct dimensions. Each ligand is color-coded in the active pocket of GABA-AT (A07 in violet, B07 in medium violet-red, D08 in blue, H08 in cyan, and vigabatrin in yellow). Hydrogen bonds, along with their bonding distances and the associated amino acid residues, are marked in red. Salt bridges are indicated in purple, while other interacting amino acid residues are shown in black. This visualization provides insight into the binding interactions of these ligands within the active site of GABA-AT.

The A07 compound, which manifested the lowest docking energy value from the selected four compounds in the molecular docking studies, followed by B07, D08, and H08, exhibited strong interaction with GABA-AT. The salt bridges and ligand–protein hydrogen bonds are shown in Figure 4. The A07 and GABA-AT docked complex expressed two salt bridges and three hydrogen bonds, which included the residues Gln329-HE, Glu293-O, Phe217-O, and Lys357. The B07–GABA-AT docked complex exhibited three hydrogen bonds with amino acids Lys357-H, Gln329-H, and Asp326-O. The D08 and GABA-AT complex interaction analysis revealed that D08 formed eight hydrogen bonds and one salt bridge with the active amino acid residues of GABA-AT, including Phe217-O, Gly219-H, Asn168-H, Asp326-O, Glu293-O, Gln329-H, and Lys357-H. The H08–GABA-AT docked complex formed three salt bridges and one hydrogen bond, including the residues Asp326, Lys357, Glu293, and Phe217-O. The vigabatrin–GABA-AT docked complex exhibited four hydrogen bonds and one salt bridge.

### 2.6. Molecular Dynamics Simulation

From the 16 docked compounds, 10 compounds based on the availability for the biological confirmation analysis were selected as A07, H08, B07, A08, H03, D08, G03, E07, and G07. These compounds exhibited comparable molecular docking energies to vigabatrin and good interactions in the molecular docking and interaction analysis, and they were further subjected to MD simulations with the reference compound vigabatrin. A 100 ns molecular dynamics (MD) simulation was conducted for each complex, and the stability of the docked complexes was assessed through various analyses, including root-mean-square deviation (RMSD), hydrogen bond plot analysis, MD interaction energy analysis, and MD binding analysis.

### 2.7. RMSD Analysis

**Figure 5:**
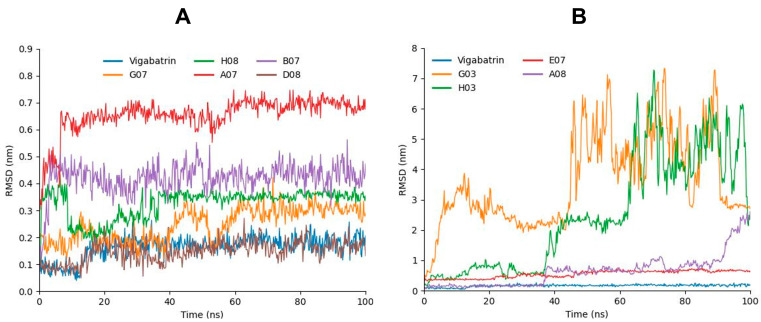
(**A**) Graph representing the RMSD analysis of A07 (red), G07 (orange), B07 (purple), H08 (green), D08 (chocolate) in comparison with vigabatrin (blue). (**B**) Graph representing the RMSD analysis of E07 (red), G03 (orange), A08 (purple), and H03 (green) in comparison with vigabatrin (blue).

By utilizing GROMACS, we conducted 100 ns long MD simulations for each complex to evaluate their flexibility and overall stability. Through the analysis of the root-mean-square deviation (RMSD) from the MD trajectories, we identified the variations in ligand positions within the active region of GABA-AT protein. A07, which manifested the lowest molecular docking energy of all respective compounds, was predicted to have relatively high RSMD values but the stability of deviations remained consistent and maintained σ = 0.60 nm RMSD values throughout the 100 ns MD simulation (Figure 5A,B). The H08 compound, which depicted a lower docking energy (−58.4782) following A07, exhibited a decrease in RMSD values at 10 ns and changed the bar line position, changed the bar line again when reaching 37 ns, and then maintained the initial RMSD values (σ = 0.38 nm) and manifested steady behavior for the rest of the 100 ns. B07, which was third in the docking studies, also exhibited a stable pattern. Although the peaks were high in the case of B07 as compared to H08, the bar line remained between σ = 0.4 and 0.45 nm throughout the 100 ns MD simulation. Interestingly, D08 and vigabatrin exhibited very similar patterns in the RMSD graph and manifested the lowest RMSD values. Moreover, as compared the top compound, E07 depicted an increasing pattern at the start, showing σ = 0.38 nm RMSD, but the bar line increased to σ = 0.65 nm at 100 ns. G03, A08, and H03 showed highly fluctuating bar lines, which depicted the lower compatibility of these compounds with GABA-AT.

### 2.8. MD Interaction Energy Analysis

**Figure 6:**
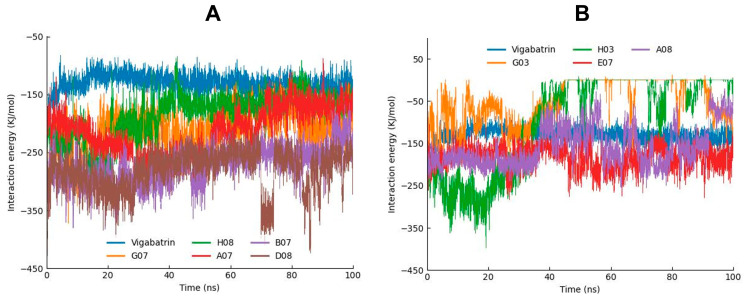
(**A**) Graph showing the MD interaction energies of A07, G07, B07, H08, and D08 in comparison with vigabatrin. (**B**) Graph showing the MD interaction energies of E07, G03, A08, and H03 in comparison with vigabatrin.

The interaction energies of all of the simulated compounds were also calculated from the MD trajectories. The interaction energy calculation was carried out in two forms: electrostatic (Coulombic) interaction energy and Lennard–Jones interaction energy, with their sum representing the total interaction energy. According to the interaction energy analysis, D08 manifested the lowest interaction energy. B07 and A07 (−273.039 and −214.573) also manifested lower interaction energies following D08. Moreover, G07 and H08 depicted low interaction energies as compared to the other compounds (Table 2). Furthermore, the interaction energies of these compounds and vigabatrin were also depicted in graphical representation to analyze the bar line patterns throughout the 100 ns MD trajectories (Figure 6A,B).

### 2.9. Hydrogen Bond Plot Analysis

In the hydrogen bond plot analysis, hydrogen bonds were categorized into two types, actual hydrogen and potential hydrogen bonds. The hydrogen bonds were observed in the trajectory of the MD simulation. During the simulation, the positions of atoms were recorded at regular intervals, and GROMACS could identify instances where a hydrogen bond formed based on predefined criteria (e.g., distance and angle criteria). If the criteria were met, a hydrogen bond was considered an “actual hydrogen bond” during that specific time step. On the other hand, the interactions that had the potential to form hydrogen bonds but did not satisfy the specific criteria for a hydrogen bond in a given time step of the simulation were referred as “potential hydrogen bonds.” This is dedicated to the situation in which the atoms involved are close to meeting the criteria but the geometry is not satisfied at that particular moment; however, potential hydrogen bonds have the potential to form a hydrogen bond if the criteria are satisfied in the upcoming frame.

**Figure 7:**
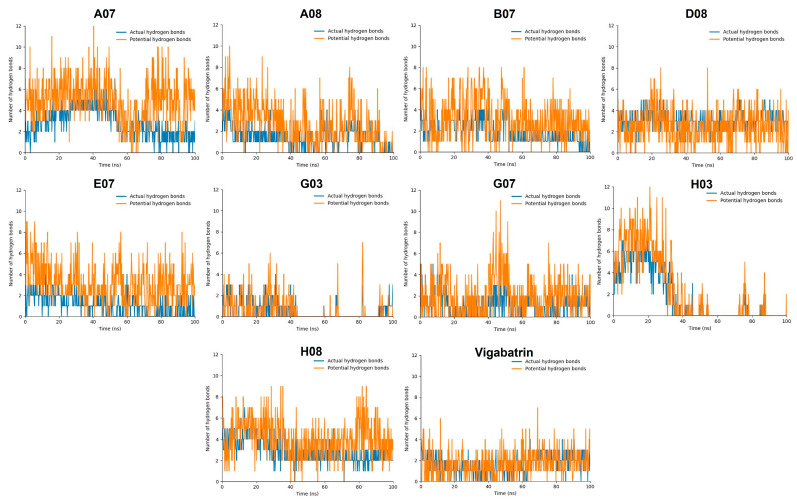
The graphs display the formation of hydrogen bonds during the 100 ns MD simulation. Actual hydrogen bonds are colored blue, while potential hydrogen bonds are colored orange.

The compounds A07, B07, D08, H08, E07, and G07 manifested good ratios of hydrogen bonds during the hydrogen bond plot analysis. All of these compounds manifested three or four potential hydrogens bonds and one or two actual hydrogen bonds at the same time, although the hydrogen bond positions kept changing. Compound D08, which manifested the most stable RMSD value, depicted a lot of potential hydrogen bonds while only 1 or 2 actual hydrogen bonds through the 100 ns MD trajectory. Furthermore, B07 and A07 also showed high numbers of potential hydrogen bonds, up to 12 bonds for A07 and 8 for B07 (Figure 7). In contrast, G03 and H03 manifested lower numbers of potential hydrogen bonds as compared to vigabatrin. Compound H03 also manifested a good ratio of hydrogen bonds at the start of the MD simulation but the number of hydrogen bonds kept decreasing and sometimes diminished with the passage of time.

The compounds A08, G03, and H03 exhibited destabilizing RMSD values as well as elevated interaction energy values in the RMSD and interaction energy analyses and were found not to be compatible in the hydrogen bond analysis. These findings suggested that these compounds may not form stable interactions with the target over the course of the MD simulations, indicating limited or unfavorable behavior in terms of binding to GABA-AT protein. Although the performance was good at the start of the MD simulation, the number of hydrogen bonds kept decreasing with the passage of time during the 100 ns MD simulation.

### 2.10. MD Binding Mode Analysis

**Figure 8:**
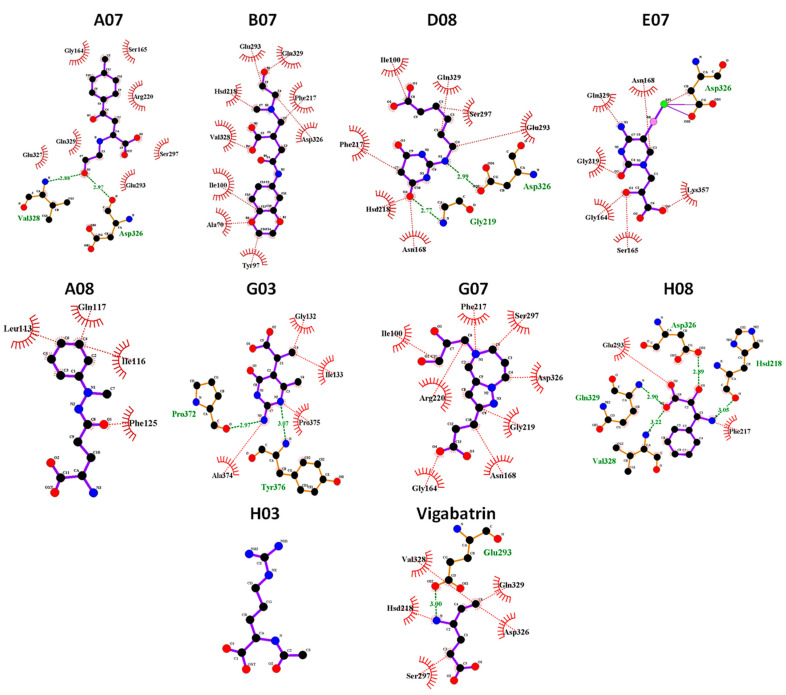
Graphical depiction of binding mode analysis. The hydrogen bonds are indicated in green dotted lines with the distance. The hydrophobic interactions were colored red. The H03 compound was not found to be in contact with the target protein at 100 ns MD.

To scrutinize the interactions of the screened compounds at the end of the 100 ns MD simulation, MD binding mode analysis was conducted. Snapshots of all nine simulated compounds, in contrast to the reference compound vigabatrin, were captured at the 100 ns mark and subsequently visualized using UCSF Chimera and LigPlot [31]. The results demonstrated that the compounds A07, B07, D08, and H08 remained in the active region of GABA-AT during the 100 ns MD simulation and maintained hydrophobic interactions and hydrogen bonds with binding pocket amino acid residues of GABA-AT (Figure 8). Moreover, the compounds G07 and E07 also maintained hydrophobic interactions with active region amino acid residues. In contrast, the compounds H03, G03, and A08, which showed highly fluctuating RMSD graphs and high interaction energies, were found to be out of the binding pocket of GABA-AT at the end of the 100 ns MD simulation.

### 2.11. gmx_MMPBSA Binding Free Energy Calculation

The complete trajectories obtained from the 100 ns MD simulations were utilized for Molecular Mechanics Poisson–Boltzmann Surface Area (MMPBSA) analysis, which aimed to investigate and calculate the binding free energy of the simulated complexes. The GROMACS tool gmx_MMPBSA was employed, and the MM/PBSA method was applied to compute the binding energy with default parameters. Consequently, the ΔG (binding free energy) and standard deviation values were determined. The average ΔG and average standard deviation values are exhibited in Table 3. The four best compounds, A07, B07, D08, and H08, showed the lowest ΔG values and had high binding affinities as compared to the reference compound vigabatrin (= 0.25ΔG). The G07 compound manifested a positive free energy value (Table 3), suggesting that it did not bind efficiently with GABA-AT.

### 2.12. Structure Evaluation, Similarity Comparison, and Common Substructure Finding

**Figure 9:**
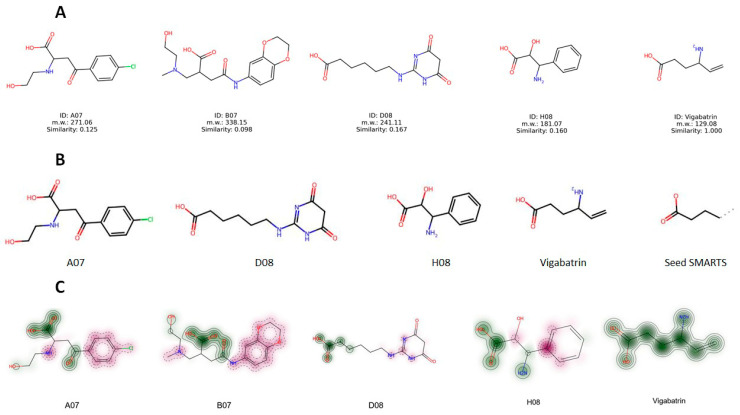
(**A**) Structures of vigabatrin and highly predicted compounds. (**B**) Common structural motif found with Maximum Common Substructure (MCS) by SMARTS. (**C**) Graphical representation of shared common structural motif by similarity maps.

The chemical structures of the screened compounds and vigabatrin are presented in Figure 9A. To assess the structural resemblance between these compounds, the Tanimoto similarity measure in RDKit was applied. The analysis showed no significant structural similarity between the screened compounds and vigabatrin, as indicated by the Tanimoto similarity coefficient (Table 4). While there is typically no specific threshold for determining similarity, a Tanimoto similarity value of 0.8 or higher can be regarded as indicative of similarity on a scale of 0 to 1. In this scale, a value of 0 denotes no similarity, whereas a value of 1 implies complete similarity. Despite the screened compounds having limited overall similarity to vigabatrin, these compounds might still share certain common structural motifs. To identify these common substructures, the Maximum Common Substructure (MCS) algorithm utilizing SMARTS (Smiles Arbitrary Target Specification) in RDKit was employed. Interestingly, as depicted in Figure 9B, a recurring structural motif, referred to as Seed SMARTS, was found in the majority of compounds with the exception of B07 (Figure 9B). This finding suggested that the recurring substructure may play a role in GABA-AT inhibition. In addition, similarity maps with fingerprints in RDKit were used to illustrate whether the screened molecules contained the structural motif of vigabatrin (Figure 9C). The similarity maps of the screened compounds exhibited the presence of the structural motif of vigabatrin in their structures. The MCS and similarity map findings provide valuable information to further design novel candidate compounds.

### 2.13. Experimental Validation of GABA-AT Inhibition

**Figure 10:**
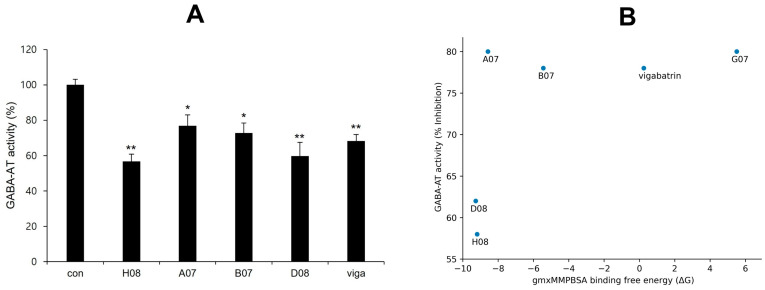
(**A**) GABA-AT inhibitory activity of screened compounds in comparison to vigabatrin. The concentration of vigabatrin and the screened compounds (H08, A07, B07, and D08) was 25 μM (single (*) and double (**) marks represent statistical significance at *p* < 0.05 and *p* < 0.01, respectively). (**B**) Scatterplot between GABA-AT inhibitory activity and gmx_MMPBSA binding free energy (ΔG).

GABA-AT inhibitory activity of screened compounds and vigabatrin was experimentally determined in U87MG glioma cells using a resazurin-based assay to measure GABA-AT activity. Glial cells express GABA-AT, and U87MG cells, which are derived from a human malignant glioma, have been utilized in diverse research related to GABA [32]. In a preliminary study, 50 compounds were selected for the GABA-AT assay in U87MG cells after being highly predicted as potential inhibitors in the pharmacophore analysis from a library of 530,000 compounds. Of these 50 compounds, 16 demonstrated equal or greater inhibitory activity than vigabatrin and were chosen for further evaluation. Ultimately, four compounds (H08, A07, B07, and D08) exhibited significant inhibition of GABA-AT. Notably, H08 and D08 showed more inhibitory potential than vigabatrin (Figure 10A). Scatterplot graph analysis was employed to explore the potential correlation between biological GABA-AT inhibitory activity and gmx_MMPBSA binding free energy (ΔG). While H08 and D08 exhibited a strong correlation, no general correlation was observed (Figure 10B). The non-linear behavior of the correlation may be attributed to the inherent differences between computational algorithms and biological evaluation.

## 3. Materials and Methods

### 3.1. GABA Structure Retrieval

GABA-AT is a homodimeric enzyme with 461 amino acid residues and a molecular weight of 56 kDa per monomer. The crystal structure of human GABA was not available in Protein Data Bank (PDB) (https://www.rcsb.org/) (accessed on 1 March 2023). The primary structure GABA-AT was deduced from our previous study, in which the cDNA of the *Sus scrofa* brain was utilized for homology modeling of the human GABA-AT 3D structure [27]. The *Sus scrofa* GABA-AT enzyme sequence had 95.67% homology with the *Homo sapiens* enzyme, which authenticated studies with the *Sus scrofa* enzyme as being highly pertinent to humans.

### 3.2. Prediction of Active Binding Site

The interacting site in the protein’s holo-structure most likely determines the binding pocket of the protein, where the active ligand binds [33]. The binding pocket residues were selected from already published data [27] as Ile100, Ser102, Ala162, Cys163, Gly164, Ser165, Phe217, His218, Gly219, Arg220, Glu293, Asp326, Val328, Gln_329, Gln330, Ser356, Lys357, and Met360. Furthermore, the binding pocket was visualized using UCSF Chimera and Discovery Studio for residual position comparison and validation.

### 3.3. Pharmacophore Modeling and Molecular Docking Analysis

To create a pharmacophore model of reasonable size, the receptor–ligand pharmacophore generation algorithm was employed. This approach generated selective pharmacophore models based on non-bond interactions between the protein and ligand. The Search 3D Database protocol in Discovery Studio was utilized to create a pharmacophore model of the vigabatrin and GABA-AT complex, and the generated pharmacophore model hit compounds from a large database were filtered, such as the 530,000 compounds in the Korea Chemical Bank (KCB). The KCB database is an indexed multi-conformer database constructed using the Build 3D Database protocol in Discover Studio [34]. The screened 50 compounds were further analyzed by preliminary biological studies. Therefore, 16 compounds were assessed for molecular docking.

Molecular docking is a widely employed method for assessing the interactions and conformations of ligands when binding to target proteins [35]. Molecular docking predicts the strength of association or binding compatibility between a ligand and protein by considering their preferred orientation and employing scoring algorithms [28]. Before starting the docking protocol, both water and the ligand molecule were removed from the receptor, and hydrogens were added using the Discovery Studio protein preparation module. Ligand preparations were also conducted for both the reference (vigabatrin) and candidate compounds. These preparations involved tautomerization, ionization state adjustments, and correction of any problematic valences, which were addressed using the Discovery Studio ligand preparation module. The molecular docking itself was performed using the Discovery Studio CDocker module. The default orientation and conformation settings were applied during the docking process. To assess the quality of the docking, the lowest CDocker interaction energy values (in kcal/mol) were used to identify the best-docked complexes.

### 3.4. Molecular Dynamic Simulation

The 10 molecules that manifested a comparable molecular docking energy (Kcal/mol) with vigabatrin and good interactions with GABA-AT in the docking interaction analysis underwent a 100 ns MD simulation experiment with the reference compound vigabatrin. The CHARMM36 force field was created using the solution builder protocol provided by the CHARMM-GUI server (https://www.charmm-gui.org) (accessed on 1 March 2023). Input files for the molecular dynamics (MD) simulations in GROMACS were generated using the same methodology and parameters [36]. For generation of the MD input file, five steps were followed. (1) In the first step, the predicted 3D structure of GABA-AT was uploaded in complex with the docked compound. (2) In the second step, the TIP3P solution was used to solvate the current model within a periodic rectangular box that extended 10 Å beyond each peptide’s atom. The ion placement method selected was the Monte Carlo method. The basic ion type was KCL, and the ion concentration was adjusted to 0.15 by default. Counter ions were added until the system reached neutralization. (3) In step three (solvator), the dimensions of the box along each axis (A, B, and C) were set to 94 Å, resulting in a total system size of approximately 830,584 cubic Angstroms. The crystal type was specified as cubic in symmetry, and the crystal angle values Alpha, Beta, and Gamma were set to 90 degrees, which is typical for cubic lattices. The box type was chosen as rectangular. Moreover, the simulation included the use of the Verlet cutoff technique with a 10 Å cutoff for both electrostatic and Van der Waals interactions. The LINCS algorithm was employed to constrain bond lengths. To compute electrostatic interactions, the particle mesh Ewald (PME) method was utilized. (4) In step four, the solvated systems underwent two equilibration phases. The NVT (constant number of particles, volume, and temperature) condition was initially applied to the systems before transitioning to the NPT (constant number of particles, pressure, and temperature) condition. The temperature for the simulation was set at 30 °C. (5) In step five, for the MD simulations using GROMACS, a format conversion Python script provided by CHARMM-GUI was used to generate GROMACS topology (top) and parameter (itp) files. GROMACS software version 2019.3 was employed on a Linux operating system to investigate the structural behavior of the protein and ligand complexes. The production dynamics were conducted with a 2 fs time step, and the coordinates were saved every picosecond for subsequent analysis.

### 3.5. gmx_MMPBSA Binding Free Energy Calculation

A program called gmx_MMPBSA was developed to calculate end-state free energies using molecular dynamics trajectory data obtained from GROMACS. Its primary purpose was to determine the binding free energies of protein–ligand complexes [37]. The MM/PBSA (Molecular Mechanics/Poisson-Boltzmann Surface Area) approach was employed for predicting binding free energies based on the molecular dynamics (MD) simulation trajectories conducted in explicit solvent. This approach calculates the binding free energy by considering three components separately: the complex (protein–ligand complex), the receptor (protein), and the ligand (small molecule) [38]. The MD simulation trajectories spanning from 0 ns to 100 ns were harnessed to compute the binding free energies for the top 5 complexes. The representation of the binding free energy (ΔG_binding_) of the lead compounds, in complex with protein, was calculated using the following equation:

ΔG_binding_ = G_complex_ − (G_protein_ + G_ligand_) (1)

In the equation mentioned above, Gcomplex represents the energy of the complex formed by the lead compound and protein. Gprotein and Gligand represent the energy of the protein and ligand, respectively, within a water environment. These terms were used to calculate the binding free energy in the MM/PBSA approach.

### 3.6. Experimental Reagents and Cell Culture

U87MG cells were purchased from the Korea Cell Line Bank (KCLB, #30014) and maintained in Dulbecco’s Modified Eagle’s Medium (DMEM; Hyclon Laboratories (Logan, UT, USA)). The medium was supplemented with 10% heat-inactivated fetal bovine serum (FBS; GIBCO, Thermo Fisher Scientific, Waltham, MA, USA) and 100 U/mL penicillin-streptomycin (GIBCO, Thermo Fisher Scientific, Waltham, MA, USA) and cultured at 37 °C under 5% CO_2_. All chemicals used in the study were obtained from the Korea Chemical Bank (KCB, http://www.chembank.org, accessed on 1 March 2023) of the Korea Research Institute of Chemical Technology (KRICT). The enzymatic reagents were purchased from Sigma (Sigma-Aldrich, Saint Louis, MO, USA).

### 3.7. Resazurin-Based Assay for GABA-AT Activity Determination

U87MG cells were cultured in a 6-well plate for 1 day. The cells were then incubated with 25 µM KCB compounds or vigabatrin for 2 days without changing the medium. After incubation, the cells were harvested in 200 µL of ice-cold lysis buffer (containing 100 mM sodium phosphate, pH 7.0, 20 mM pyridoxal phosphate, and 0.1% triton X-100). The lysate was freshly prepared before the enzyme activity assay. The GABA-AT activity assay was performed as previously described [39,40]. Briefly, GABA-AT activity was determined using a coupled succinic semialdehyde dehydrogenase method. For this measurement, 10 µL of cell lysate was combined with 190 µL of master mix, consisting of 0.063 U/mL diaphorase, 6.25 mM resazurin, 1 mM nicotinamide adenine dinucleotide hydrate (NAD), 5 mM alpha-ketoglutarate, 3.5 mM mercaptoethanol, and 6 mM GABA in 100 mM potassium pyrophosphate buffer (pH 8.6). The master mix was prepared fresh before the experiments. Next, 10 µL of lysate was dispensed into a 96-well clear bottom black plate (Coster, South Elgin, IL, USA), followed by the addition of 190 µL of master mix. The reaction occurred at room temperature and was shielded from light for 30 min. The fluorescence was quantified using a microplate reader with an excitation wavelength of 544 nm and emission wavelength of 590 nm (Molecular Device, SpectraMax, M5, San Jose, CA, USA).

### 3.8. Statistical Analysis

All values shown in the figures are expressed as the mean ± SD obtained from at least three independent experiments. Statistical significance was analyzed using a two-tailed Student’s *t*-test. Data with values of *p* < 0.05 were considered statistically significant. Single (*) and double (**) marks represent statistical significance at *p* < 0.05 and *p* < 0.01, respectively.

## 4. Conclusions

GABA plays a critical role in neurotransmitter inhibition in the brain and is a significant target for drugs used in the treatment of neurological disorders such as epilepsy. GABA-AT is a target for such drugs, as inhibiting GABA-AT can lead to an increase in GABA levels in the brain. In our study, we utilized various computational methods to identify novel and highly potent inhibitors of human GABA-AT that surpassed the known inhibitor, vigabatrin, in terms of efficacy. Our research identified several promising compounds, such as A07, B07, D08, and H08, which exhibited results closely comparable to those of vigabatrin. These compounds demonstrated favorable characteristics, including low RMSD values, the lowest binding energies in MD simulations, and high binding affinity as determined by MMPBSA free energy calculations. These findings suggest that these compounds could serve as potential therapeutic options for targeting GABA-AT and addressing neurological disorders associated with GABA deficiency in the brain.

## Figures and Tables

**Table 1:** The docking energy values (in kcal/mol) for the screened compounds when docked with GABA-AT protein were determined using Discovery Studio. These values reflect the calculated binding energies of the compounds to GABA-AT protein and are crucial for assessing their potential as inhibitors or ligands for GABA-AT.

Compounds	CDocker Energy	CDocker Interaction Energy
D07	−72.6	−75.1
F07	−72.1	−71.2
B08	−60.5	−53.4
A07	−59.4	−57.3
H08	−58.5	−53.3
B07	−57.4	−60.5
C08	−56.8	−56.3
A08	−56.6	−52.7
H03	−55.3	−55.6
Vigabatrin	−53.7	−56.3
D08	−52.2	−54.6
G03	−49.1	−38.1
G06	−46.6	−59.5
E07	−44.8	−47.9
E04	−38.5	−48.7
G07	−30.9	−51.1
H06	−17.6	−41.4

**Table 2:** The interaction energies of all nine compounds in comparison with vigabatrin.

Compounds	Coul-SR	LJ-SR	Total
G03	−22.7	−29.2	−51.9
H03	−59.1	−37.6	−96.8
A07	−101.4	−113.1	−214.6
B07	−151.9	−121.1	−273.0
G07	−83.1	−126.9	−210.0
E07	−89.6	−92.4	−182.0
A08	−81.2	−77.6	−158.8
D08	−174.6	−106.7	−281.2
H08	−116.4	−69.6	−186.0
Vigabatrin	−62.6	−68.3	−130.9

**Table 3:** The binding affinities of the top 5 simulated compounds.

Sr	Compounds	ΔG_(TOTAL)_	Standard Deviation
1	A07	−8.58	8.02
2	B07	−5.44	5.12
3	D08	−9.27	5.87
4	H08	−9.19	6.04
5	G07	5.51	7.19
6	Vigabatrin	0.25	4.23

**Table 4:** Tanimoto similarity comparison of vigabatrin and screened compounds. The Tanimoto similarity of each compound was calculated compared to the other compounds.

Similarity	A07	B07	D08	H08	Vigabatrin
A07	-	0.275	0.157	0.217	0.125
B07	0.275	-	0.152	0.159	0.098
D08	0.157	0.152	-	0.079	0.167
H08	0.217	0.159	0.079	-	0.160
Vigabatrin	0.125	0.098	0.167	0.160	-

## Data Availability

All the data are available in the manuscript and the supplementary data.

## Supplementary Materials

The following supporting information can be downloaded at: https://www.mdpi.com/article/10.3390/ijms242316990/s1.
