# Supplementary material for: Discovery of GABA Aminotransferase Inhibitors via Molecular Docking, Molecular Dynamic Simulation, and Biological Evaluation
Source: Int J Mol Sci. 2023 Nov 30;24(23):16990. doi: 10.3390/ijms242316990 (PMC10707509; doi:10.3390/ijms242316990)
Supplement: Supplementary file 1 [file ijms-24-16990-s001.zip › ijms-2738556-supplementary.pdf]

Discovery of GABA aminotransferase Inhibitors via Molecular Docking, Molecular Dynamic Simulation, and Biological Evaluation

Muhammad Yasir[1], Jinyoung Park[1], Yuno Lee[2], Eun-Taek Han[3], Won Sun Park[4], Jin-Hee Han[3], Yong-Soo Kwon[5], Hee-Jae Lee[1], and Wanjoo Chun[1,*]

[1]Department of Pharmacology, Kangwon National University School of Medicine, Chuncheon, 24341, Republic of Korea.

[2]Drug Information Platform Center, Korea Research Institute of Chemical Technology, Daejeon, 34114, Republic of Korea.

[3]Department of Medical Environmental Biology and Tropical Medicine, Kangwon National University School of Medicine, Chuncheon, 24341, Republic of Korea.

[4]Department of Physiology, Kangwon National University School of Medicine, Chuncheon, 24341, Republic of Korea.

[5]College of Pharmacy, Kangwon National University School of Medicine, Chuncheon, 24341, Republic of Korea.

Corresponding author: Dr. Wanjoo Chun, Department of Pharmacology Kangwon National University School of Medicine, Kangwon National University, Email: wchun@kangwon.ac.kr, Phone: +82-33-250-8853.

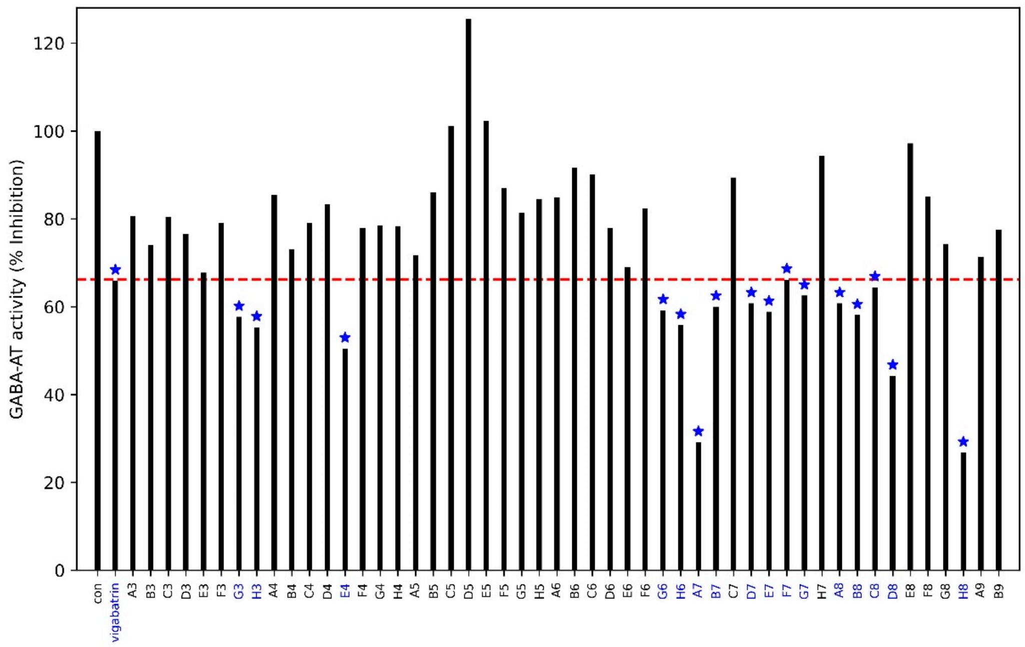

Figure S1. Preliminary GABA-AT inhibitory activity screening of pharmacophore-filtered KCB 50 compounds

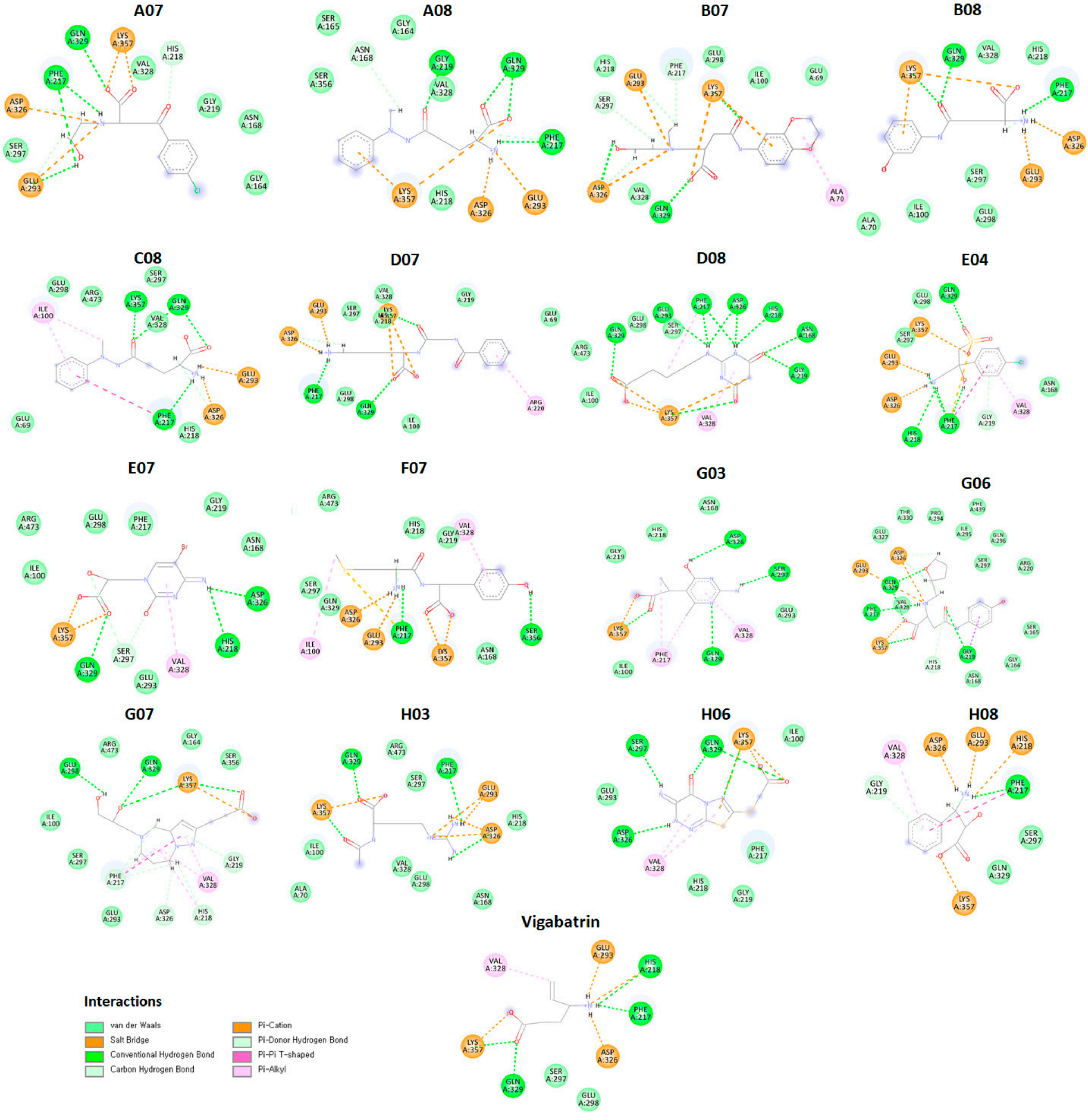

Figure S2. The interaction analysis of sixteen docked complexes.
